# Exploring glycocalyx components powering the contact between *Entamoeba histolytica* and bacteria during non-opsonic phagocytosis

**DOI:** 10.1371/journal.ppat.1014560

**Published:** 2026-09-21

**Authors:** Elisabeth Labruyère, Thomas Treport, Jean-Christophe Olivo-Marin, Nancy Guillen

**Affiliations:** Institut Pasteur, Université Paris Cité, Biological Image Analysis Unit, Centre National de la Recherche Scientifique UMR3691, Paris, France; University of Queensland, AUSTRALIA

## Abstract

Phagocytosis is a cornerstone of the immune defence, allowing cells to eliminate pathogens through both opsonic and non-opsonic pathways. In protozoa such as *Entamoeba histolytica*, non-opsonic phagocytosis is not only central to survival but also to its ability to cause disease, with its life cycle closely intertwined with the intestinal microbiota, which supplies bacteria and their metabolic byproducts to support trophozoite growth. Yet, despite its significance, the molecular basis of how *E. histolytica* recognises and engulfs bacteria remains largely unresolved. This review examines the cell-surface molecules that may mediate bacteria–amoeba interactions and highlights new strategies to uncover their roles, offering fresh insight into the dynamics of non-opsonic phagocytosis.

## 1. Bacteria non-opsonic phagocytosis by amoebae

Phagocytosis is a fundamental cellular process by which cells eliminate pathogens, clear infections, and activate host defence mechanisms. This process is conserved across a wide range of organisms, from unicellular protozoa to complex multicellular animals, including humans. Cells specialised in phagocytosis known as phagocytes—such as macrophages, dendritic cells, and neutrophils—are adept at recognising, internalising, and degrading bacteria, cellular debris, and other foreign substances [1].

Phagocyte recognition of microorganisms occurs through two main mechanisms: opsonic phagocytosis, in which host-derived opsonins—such as antibodies and complement proteins—bind to the surface of pathogens, tagging them for recognition by specific receptors on the phagocyte (e.g., Fc receptors and complement receptors). Conversely, non-opsonic phagocytosis involves direct interactions between surface molecules on the phagocyte and molecular motifs on the microorganism, such as key non-opsonic receptors that recognises Pathogen-Associated Molecular Patterns (PAMPs) (e.g., scavenger receptors, C-types lectins) [2].

Although both forms of phagocytosis are necessary for immune functions, non-opsonic phagocytosis is particularly significant in vital activities of invertebrates and unicellular eukaryotes [3], highlighting its evolutionary importance. For example, in protozoa, bacterial phagocytosis is inherently non-opsonic and is essential for their survival, as it allows them to feed on microorganisms in a wide range of environments, including aquatic systems, soil, sediments, and the gastrointestinal tract [4]. Among protozoa, amoebae are particularly noteworthy due to their predation on microbial communities as a primary source of nutrients [5]. While numerous studies have explored bacteria–amoebae associations, the complexity of these interactions remains underexplored. Much of the current focus has been on free-living amoebae, where phagocytosed bacteria can evolve strategies to evade degradation, persist, and even replicate within the intracellular amoebic environment. This is exemplified by *Acanthamoeba* spp., which serve as environmental reservoirs for a variety of pathogenic bacteria [6].

## 2. *Entamoeba histolytica* feeding on the microbiota

Species of the *Entamoeba* genus are highly proficient phagocytes; seven of these species are known to infect humans and rely on the intestinal microbiota as a primary nutrient source. *Entamoeba histolytica* is of particular interest, as it is the causative agent of amoebiasis, a globally prevalent infectious disease [7]. In the human colon, *E. histolytica* exhibits a relatively simple life cycle that alternates between two cellular distinct forms: the trophozoite and the cyst. Both stages are strongly influenced by the intestinal microbial environment, which not only supplies bacteria and their metabolic byproducts to support trophozoite growth but also acts as a key driver of encystation—an essential process for parasite survival under adverse conditions. The interaction between bacteria and *Entamoeba* is therefore central to the parasite’s survival and proliferation within the gut lumen.

Extensive bacteria phagocytosis contributes to dysbiosis [8], thereby fostering an environment conducive to parasite proliferation [9]. Bacteria phagocytosis by *E. histolytica* is primarily determined by nutrient acquisition, competition within the intestinal ecosystem, and parasite virulence, rather than by bacterial pathogenicity. Indeed, the pathogenic parasite *E. histolytica* phagocytises bacteria more efficiently than non-pathogenic *Entamoeba* species but does not selectively target pathogenic bacteria [7]. The hypothesis that *E. histolytica* preferentially phagocytises certain bacteria present in the gut microbiota has been investigated [8]. Metagenomic analysis of the bacterial population isolated from stools of healthy individuals has shown that upon contact with *E. histolytica* certain beneficial bacteria are preferentially ingested, including *Lactobacillales, Erysipelotrichales, Clostridales, and Bifidobacteriales* [8]*.* The exact mechanisms and specificity for phagocytosis of different bacteria are still being researched. Despite its biological significance, the molecular and cellular mechanisms underlying this microbial interspecies interaction remain poorly understood. In particular, the processes that mediate bacteria recognition by *E.histolytica* and the subsequent activation of non-opsonic phagocytosis have not been fully elucidated.

*A fundamental question persists: How do Entamoeba histolytica and bacterial surfaces recognise each other, and what molecular events trigger phagocytosis?*

This review synthesises current knowledge of *Entamoeba histolytica* interactions with bacteria, with a particular focus on the molecular components underlying non-opsonic phagocytosis. We propose a mechanistic model in which specific surface-associated molecules of *E. histolytica* act as bacterial recognition receptors, mediating the selective binding of bacterial surface ligands. This receptor-driven framework suggests that bacterial recognition and internalisation are tightly coupled processes governed by defined host–microbe molecular interactions. Based on this model, we propose experimental approaches to identify the receptors, ligands, and downstream signalling pathways that orchestrate *E. histolytica*–bacteria interactions.

## 3. Bacteria surface components recognised by phagocytes

A central determinant of bacteria and diverse cell types of interactions is the glycocalyx. It is the outermost layer of cells composed of a cross-linked, gelatinous network of glycans covalently attached to proteins or lipids [10] which extraordinary complexity stems from the structural diversity of carbohydrates [11], positioning glycans as central determinants of host-pathogen interactions [12]. Several well-known bacterial surface components are involved in interactions with eukaryotic cells; these can be grouped into (1) protein-anchored glycans, (2) glycopolymers, (3) lipid-anchored glycans, and (4) lectins.

### 3.1. Protein-anchored glycans as peptidoglycan (PGN)

It is a rigid polymer made of alternating N-acetylglucosamine (GlcNAc) and N-Acetylmuramic acid (MurNAc) residues cross-linked by peptides [13]. PGN provides structural support, serves as a scaffold for adhesins, and stimulates host immune responses through recognition by pattern recognition receptors [13].

### 3.2. Capsular polysaccharides (CPS)

These are composed of glycopolymers that form a capsule on the surface of many bacterial species [14]. In Gram-positive bacteria, CPS is covalently attached to the thick PGN layer. In Gram-negative bacteria, CPS is associated with outer-membrane components and capsule-retention proteins [15]. CPS generally reduces bacterial adhesion but can also function as an adhesin. Some capsules contain sialic acid, which mimics host glycans and facilitate binding to cell receptors [16].

### 3.3. Lipid-anchored glycans

The bacterial glycolipids candidates for be involved in non-opsonic phagocytosis include lipopolysaccharides (LPS), wall teichoic acids (WTA), and lipoteichoic acids (LTA). LPS, characteristic of Gram-negative bacteria [17], consists of Lipid A, which anchors LPS to the outer membrane; a conserved core oligosaccharide, and the highly variable O-antigen, responsible for serotype diversity [18]. WTA and LTA - from Gram-positive bacteria- are negatively charged glycopolymers composed of repeating glycerol- or ribitol-phosphate units. WTA is attached to PGN, whereas LTA is membrane-anchored through glycolipids [19].

### 3.4. Bacterial surface lectins

These glycoproteins facilitate bacterial adhesion by recognising specific host cells glycans [20]. In both Gram-negative and Gram-positive bacteria, lectin activity is often mediated by adhesins located on pili and fimbriae, which bind host glycoproteins and glycolipids [21]. Examples include: FimH, located at the tip of type 1 pili, which specifically binds mannose residues [22]. PapG, found on P pili, which recognises host glycans [23].

### 3.5. Eukaryotic cell surface components involved in bacterial recognition

In professional phagocytes, numerous cell surface molecules have been identified that function as pattern recognition and adhesion receptors, mediating bacterial binding and non-opsonic phagocytosis. These receptors recognise a wide range of bacterial surface determinants, including glycans, glycolipids, lipoproteins, and adhesins, thereby coupling microbial recognition to the signalling events required for particle internalisation.

The diversity of these recognition systems highlights the existence of multiple, partially overlapping mechanisms for bacterial uptake. Below, we provide a brief overview of receptor classes that may be relevant to bacterial recognition and phagocytosis by *E. histolytica*. Sialic acid-binding immunoglobulin-like lectins (Siglecs) recognise bacterial sialylated glycans [24], whereas Toll-like receptors (TLRs), particularly TLR4, detect conserved bacterial molecules such as LPS [25]. Scavenger receptors, including SR-A1, MARCO, and CD36, bind a broad spectrum of bacterial lipids and cell surface components and contribute directly to bacterial internalisation [26]. Similarly, the G protein-coupled receptor BAI1 functions as a pattern recognition receptor that directly binds LPS and promotes the phagocytosis of Gram-negative bacteria [27]. Additional carbohydrate-recognition systems participate in bacterial uptake: surface-exposed calreticulin recognises microbial glycans and enhances phagocytosis [28], while galectins bind bacterial carbohydrate motifs present in LPS and other glycoconjugates [29,30]. Furthermore, carcinoembryonic antigen-related cell adhesion molecules (e.g., CEACAM3) mediate non-opsonic phagocytosis through the direct recognition of bacterial adhesins [31]. Collectively, these findings demonstrate that eukaryotic cells employ a diverse repertoire of surface receptors to interact with bacterial ligands and initiate phagocytic uptake. This conceptual framework provides a useful basis for investigating whether analogous receptor systems and recognition mechanisms operate in *E. histolytica*.

## 4. Glycosylation pathways in *E. histolytica*

A functional glycocalyx is essential for *E. histolytica* during bacterial phagocytosis, interaction with mucus, and the self-aggregation process leading to encystation [7]. Over the last decade, great progress has been made toward the characterisation of individual glycocalyx components namely N-glycans and O-glycans on glycoproteins, glycolipids, and glycosaminoglycans (GAGs) [12].

Glycans—including those incorporated into surface molecules—are synthesised in the endoplasmic reticulum (ER) and the Golgi apparatus. They are covalently attached to proteins (forming glycoproteins and proteoglycans) or lipids (forming glycolipids) through the coordinated actions of glycosidases and glycosyltransferases [11,12].

Glycans link to proteins primarily via two mechanisms:

### 4.1. N-glycosylation

N-glycosylation involving the attachment of a lipid-linked oligosaccharide (LLO) core, composed of N-acetylglucosamine (GlcNAc) and mannose (Man), to the amide nitrogen of asparagine residues in a protein (within the Asn-X-Ser/Thr sequon, where X ≠ Pro). In eukaryotic cells, the process begins on the cytosolic face of the ER, where the lipid carrier (dolichol phosphate) is anchored. Glycosyltransferases sequentially add sugar residues to dolichol phosphate, forming the initial core (Man_5_GlcNAc_2_) that is flipped to the luminal side of the ER by a flippase, where additional Man and Glc residues are added, forming the full precursor Glc_3_Man_9_GlcNAc_2_ which is transferred by the oligosaccharyl transferase to the nascent protein.

*E. histolytica* is missing many of the glycosyltransferases that make lipid-linked precursors to N-glycans leading to accumulation of GlcNAc_2_Man_5_ core which is transferred to proteins [32]. The simplified N-glycosylation pathway of *E. histolytica* is particularly remarkable since the extensive accumulation of unprocessed Man_5_GlcNAc_2_ on mature surface glycoproteins distinguishes *E. histolytica* from most eukaryotes, which typically further process oligomannose N-glycans in the Golgi apparatus. This feature is not unique to *E. histolytica* and occurs in another anaerobic protist with reduced N-glycosylation pathways, such as *Trichomonas vaginalis* [33].

### 4.2. O-glycosylation

O-glycosylation attaches glycans, typically N-acetylgalactosamine (GalNAc), to the hydroxyl oxygen of serine or threonine residues, primarily in the Golgi apparatus. Additional sugars (e.g., sialic acid (NeuAc), Gal) are then added by glycosyltransferases forming core structures (e.g., Core 1–8 in mucin-type O-glycans). O-glycosylation exists in *E. histolytica*, although it differs substantially from the classical mucin-type O-glycosylation found in animals and the enzymes responsible for O-glycosylation in *E. histolytica* remain largely unknown but glycoconjugates with O-linked glucose-containing glycans and O-phosphodiester-linked glycans has been identified [34,35].

Thus, although unusual, the abundance of GlcNAc_2_Man_5_ (an N-glycan containing mannose residues) and the presence of non-canonical O-glycosylated components on the surface of *E. histolytica* offer interesting avenues for elucidating the mechanisms by which this parasite recognises bacteria.

## 5. Glycoconjugates at the *E. histolytica* surface

**Fig 1:**
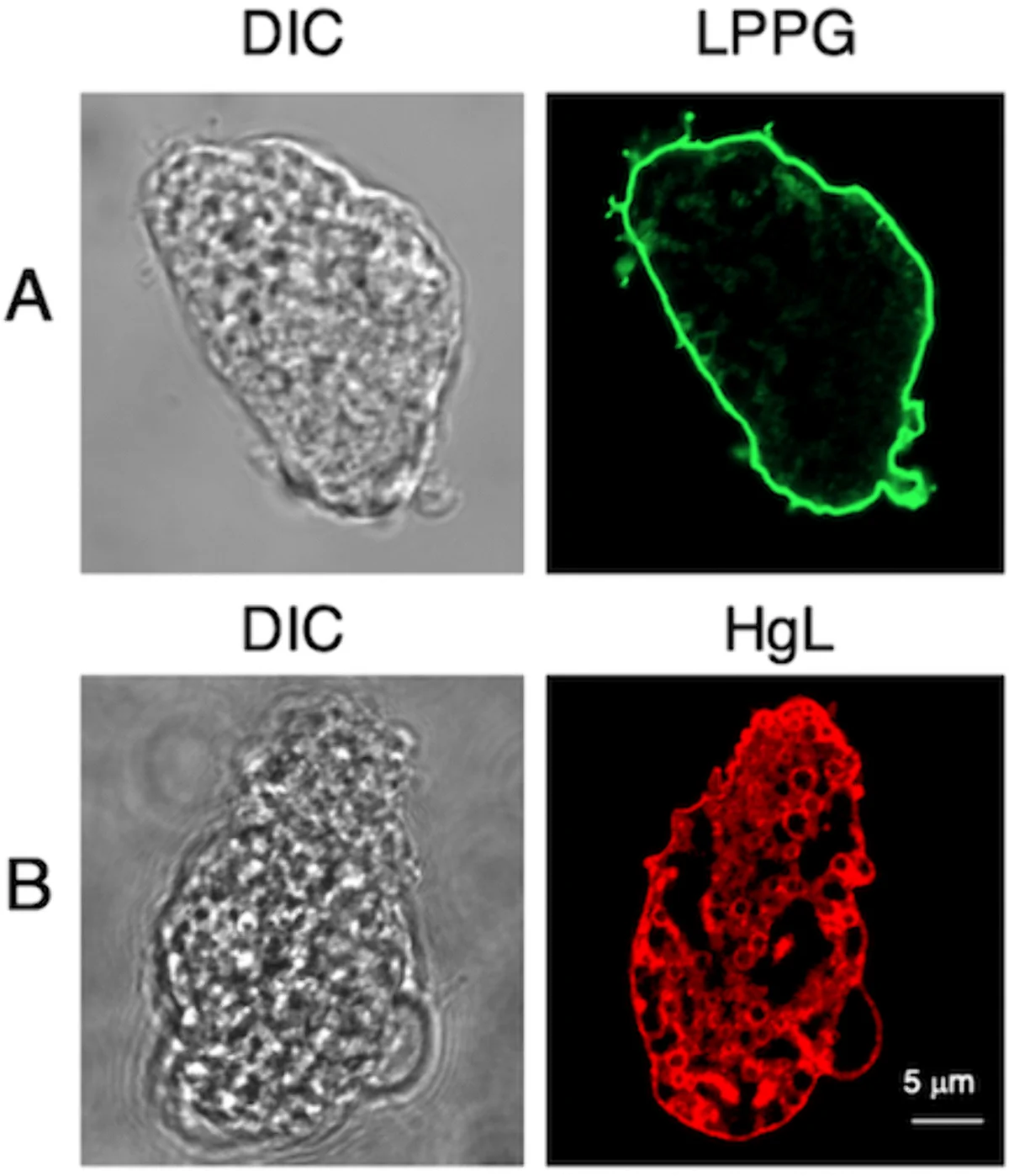
Lipopeptidophosphoglycan and the Gal-GalNAc lectin of *Entamoeba histolytica.* Growing trophozoites of the *E. histolytica* HM1-IMSS strain were fixed and prepared for confocal microscopy. Anti-LPPG (EH5 [35]) and anti-Hg subunit (7F4 [36]) monoclonal antibodies were used for indirect immunofluorescence. The left panels show differential interference contrast (DIC) images, and the right panels show immunofluorescence micrographs. Confocal sections are presented: LPPG (green) and Gal-GalNAc lectin (red), which is present in the trophozoite cytoplasm—primarily associated with vesicles—and is enriched at the surface of *E. histolytica*. Scale bar: 5 µm.

The N- and O-glycosylation pathways in *E. histolytica* remain poorly characterised compared to those of model organisms, even though heavily glycosylated factors are present on the parasite's surface. Among these, lipopeptidophosphoglycan and the Gal/GalNAc lectin are the most abundant (Fig 1), alongside glycolipids and membrane proteins. We propose below several plausible mechanisms by which these molecules, when present in the glycocalyx, could recognise bacteria.

### 5.1. Glycolipids on the surface of *E. histolytica* potentially involved in non-opsonic phagocytosis

The functions of glycolipids in bacterial recognition by *E. histolytica* remain largely unexplored. Ceramide metabolism is essential for both parasitism and encystation [37,38], and glycosphingolipids -simple glucosylceramide and lactosylceramide- has been identified [39,40]. Despite the presence of sphingolipid biosynthetic pathways, surface-expressed glycosphingolipids have not yet been identified, and no glycoglycerolipids have been reported in any *Entamoeba* species. Regarding glycerophospholipids, phosphoinositide derivatives such as PI3P and PI4P regulate phagocytosis and cyst formation, thereby highlighting their role in signalling [41]. However, surface-exposed glycolipids are expected to play a role in the internalisation of bacteria by *E. histolytica*; for example, lactosylceramide binds directly to certain pathogen-specific molecules through carbohydrate-carbohydrate interactions (e.g., β-glucans from *Candida albicans* or mycobacterial lipoarabinomannan) [42].

### 5.2. The lipopeptidophosphoglycan from *E. histolytica*

The lipopeptidophosphoglycan (LPPG) of *E. histolytica* possesses a lipid anchor (of the phosphatidylinositol type) linked to a glycan portion typical of glycolipids, composed of phosphocholine, galactose, N-acetylglucosamine, and mannose. The GPI anchor has a unique glycan core containing the sequence Gal_1_Man_2_GlcN-myo-inositol [43]. In addition, LPPG has also been considered a glycoprotein because it contains a protein core rich in serine and threonine [43,44] that is modified with linear glycans of the general structure (Glcα1–6)_n_Glcβ1–6Gal. The chains with 3–5 glucose (Glcα1,6)_n_ units, adopt a dextran-like (α-glucan) conformation [44]. Thereby, LPPG can be considered a hybrid glycoconjugate that carries extensive O-glycosylation. LPPG coats the trophozoite surface (Fig 1A), the folded structure and chemical composition suggest that bacterial adhesins—particularly those with dextran- or mannose-binding capacity—can recognise it. According with this hypothesis we propose the following bacterial factors as candidates for α-glucan (dextran) recognition within LPPG.

**a) Adhesins** containing the PA14 domain originally identified in *Bacillus anthracis* Protective Antigen. PA14 is a carbohydrate-binding module present in glycosidases, glycosyltransferases, amidases, proteases, and toxins [45]. It is common in adhesins of Gram-negative bacteria [46–48]. PA14 binds dextran in a calcium-dependent manner and mediate bacterial attachment to extracellular polysaccharides, carbohydrate substrates, or surface glycolipids/glycoproteins on various cell types [45].

**b) Glucan-binding proteins** (Gbps) or Antigen I/II present in Gram-positive bacteria such as *Streptococcus* spp. Gbps bind glucans and LTA, facilitating bacterial aggregation and biofilm development [49,50].

**c) Galactose binding adhesins.** Galactose is found in the core structure of LPPG: Gal_1_Man_2_GlcN–myoinositol glycan [42]. The terminal Gal can be recognised by adhesins such as LecA from *P. aeruginosa* or PapG *from E. coli* [51,52].

**Fig 2:**
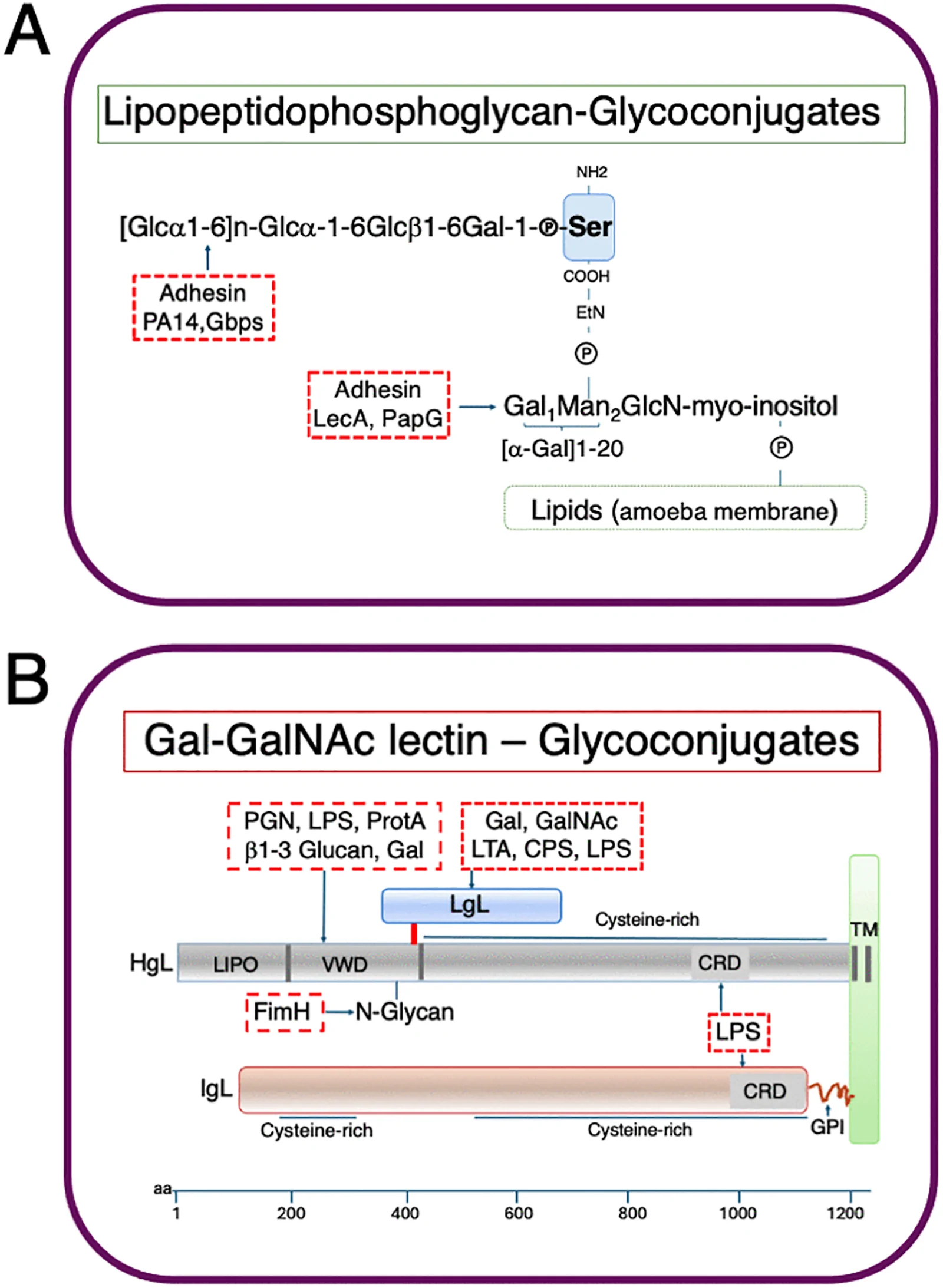
Surface molecules potentially involved in bacterial recognition by *E. histolytica.* The model illustrates how microbial interactions depend on surface molecules such as glycoprotein, glycolipids, carbohydrate recognition, and defined protein-binding domains. Potential bacterial components interacting with LPPG or the Gal-GalNAc lectin subunits are highlighted. **A) LPPG diagram.** Based on structural analyses [42], LPPG is composed of an acidic polypeptide backbone (blue square) with α1–6-linked glucans attached via phosphodiester bonds to serine residues (O-glycosylation). Anchored to the amoebic membrane via a GPI anchor containing a core glycan and α-Gal side chains. Proposed bacterial interactors of LPPG are indicated in red dashed boxes. **B) Gal-GalNAc lectin diagram.** According to recent structural data [53] the 260 kDa lectin comprises: (1) LgL (blue) with β-trefoil fold, (2) HgL (grey) containing a carbohydrate-recognition domain (CRD), N-terminal lipoprotein-like (LIPO) domain, an Asp390-linked N-glycan, and von Willebrand-like (VWD) domain; and (3) a non-covalently associated IgL (pink) bearing a CRD and a GPI anchor. LgL-Cys75 is covalently linked to HgL-Cys415 (central red line). Subunits are not drawn to scale, though approximate amino acid positions (aa) from the N-terminus are indicated. Proposed bacterial interactors are indicated in dashed boxes. Additional details are provided in the main text and Table 1.

Globally, domains of bacterial adhesins containing PA14 (Gram-), Gbps dextran-binding (Gram+), or these with Gal binding-abilities -upon interaction with LPPG- are expected to trigger signals for bacteria phagocytosis (Fig 2A). This hypothesis can be experimentally tested in binding assays using purified bacterial components and LPPG; and further, with trophozoites in the presence of bacteria carrying deficient mutant versions of the proposed adhesins.

### 5.3. Mannose-rich proteins in *E. histolytica* candidates for receptors of bacterial adhesins

*E. histolytica* exhibits selective phagocytosis of bacteria, engulfing *Escherichia coli* O115—which expresses mannose-binding type I pili—but not bacteria lacking these pili, such as *Bacteroides fragilis*, *Staphylococcus aureus*, or non-fimbriated *Shigella spp* [54]. The bacteria mannose-binding capability involved in phagocytosis was demonstrated by transfecting *Shigella flexneri* (variant strain lacking type I pili) with a plasmid encoding type I pili components. The transfected strain was efficiently attached to and phagocytosed by *E. histolytica*, whereas the isogenic non-transfected variant strain was not [55]. Bacterial attachment is inhibited by α-methylmannoside competitor for mannose binding [54]. These results suggest that the FimH adhesin of type I pili recognises mannose-rich components on the *E. histolytica* surface (Fig 2B). Furthermore, N-glycans in *E. histolytica* membranes include Man_5_GlcNAc_2_ and N-glycans containing Gal and Glc [32]. Commercial lectins exhibiting high affinity for N-glycans have enabled the identification of *E. histolytica* surface N-glycosylated proteins [56], these constitute a solid panel of glycoproteins that might interact with FimH [57]. The data indicate that FimH fulfil criteria for bacterial attachment to *E. histolytica* in order to induce phagocytosis. Direct biochemical evidence regarding the binding mechanisms of FimH to amoebic surface components remains to be determined.

### 5.4. Lectins on the surface of *E. histolytica*: the Gal/GalNAc lectin

Galactose inhibits phagocytosis suggesting that galactose-sensitive lectins mediate this phenomenon. The Gal/GalNAc lectin is the best-characterised adhesin which is enriched in the surface of *E. histolytica* (Fig 1B). This 260 kDa protein complex comprises a heavy subunit (HgL, ~170 kDa) containing a carbohydrate recognition domain (CRD) and a light subunit (LgL, ~35/31 kDa) [58]. Recent structural studies [53] revealed that HgL also contains an N-terminal lipoprotein-like domain, an N-linked glycan at Asp390, and a von Willebrand-like domain (VWD) that, in mammalian cells, binds glycoproteins and collagen. LgL is covalently linked to HgL via a disulfide bond (Cys75–Cys415) and adopts a β-trefoil 3D structure, mediating Gal/GalNAc binding. An intermediate subunit (IgL, ~150 kDa) - GPI-anchored- interacts with the HgL–LgL dimer. Multiples Cysteine rich motifs and a CRD homolog to CRD from human Galectin-2 are present in IgL [58]. The entire lectin localises to lipid rafts, facilitating adhesion to mammalian cells and downstream signalling [59].

Based on these structural data, we propose modalities for the Gal/GalNAc lectin subunit specific interactions with bacterial surface components as follow:

**a) Recognition of bacterial glycoconjugates by LgL**

Bacterial Gal or GalNAc-containing glycoconjugates are found in the core and O-antigen of LPS (*E. coli O157*, *Klebsiella pneumoniae*); in glycan units of CPS (*Streptococcus pneumoniae* serotype 14, *Staphylococcus aureus* serotypes 5 and 8); and, in LTAs (*Streptococcus agalactiae*, *Lactobacillus plantarum*). LgL subunit is predicted to recognise these bacterial glycoconjugates though its β-trefoil folding in which the Gal/GalNAc-binding site has been identified [53].

**b) Recognition of bacterial glycoconjugates by HgL**

VWD domain caries by HgL is exposed to the extracellular milieu and can participate in bacterial recognition. A role for VWD in non-opsonic phagocytosis has been demonstrated in arthropods (e.g., *Macrobrachium nipponense*) [60]. VWD-containing proteins (bind and agglutinate bacteria in a Ca^2+^-dependent manner, promoting phagocytosis by haemocytes. Purified VWD interacts with LPS, PGN, D-galactose, D-mannan, and β-1,3-glucan [60]. Evidence for the involvement of VWD in phagocytosis also comes from *Staphylococcus aureus* Protein A, which binds VWD both in suspension and when immobilised on surfaces [61]. Moreover, HgL recognises carbohydrates through the CRD domain, and the N-linked glycan is a target for bacterial adhesins.

**c) Recognition of bacterial glycoconjugates by IgL**

The CRD-like regions of IgL share homology with human Galectin-2 [58], supporting a role in recognising LPS, as in the case of human galectins with *Helicobacter pylori* LPS [29]. Other proteins may act as a co-receptor powering activities of the Gal/GalNAc lectin; one of these is Calreticulin which in *E. histolytica* contributes to amoebic antibacterial defence [62].

In summary: LgL (β-trefoil) binds to bacterial glycoconjugates containing Gal/GalNAc, HgL (VWD/ CRD/ N-linked glycan) engages with carbohydrates and glycans, and IgL (CRD-like domain) interacts with glycoproteins (Fig 2B).

### 5.5. Membrane associated proteins in *E. histolytica* with a potential role in non-opsonic phagocytosis

Although our first efforts through comparative bioinformatics analyses revealed that mammalian receptors such as Siglecs, CEACAMs, and scavenger receptors—key mediators of bacterial recognition—are absent from the *E. histolytica* proteome. However, several membrane-linked proteins stand with potentialities to be involved in non-opsonic phagocytosis in *E. histolytica*.

**a) G-protein-coupled receptor-1** (EhGPCR-1) is involved in phagocytosis and binds bacterial LPS [63]. This process is sensitive to suramin, an inhibitor of GPCR signalling. EhGPCR-1 possess seven-transmembrane helix but is not a canonical GPCR and is rather closely related to TMEM181 which recognises the cytolethal distending toxins (CDT) secreted by many pathogenic bacteria [64]. EhGPCR-1 can play functions in phagocytosis and/or sensitivity to bacteria.

**b) Transmembrane Kinases** (TMKs) comprises 80 members [65] each containing an N-terminal signal peptide, an extracellular domain with CXXC motifs (like EGF-like domains), a single transmembrane region, and a cytosolic serine/threonine kinase-like domain. Extracellular domains bear similarity to the intermediate subunit (IgL) of the Gal/GalNAc lectin [58]. In example, TMK-39 participate in *E. coli* recognition and acts as a cholesterol scavenger receptor for low-density lipoproteins [66].

**c) Leucine-Rich Repeat (LRR)** proteins often participate in immune modulation [67]. *E. histolytica* genome encodes a large family of LRR proteins [68] whose hallmark is their homology with the Bacteroides surface protein A (BspA), involved in adhesion to extracellular matrices in *Bacteroides forsythus* and *Trichomonas vaginalis*. Certain LRR proteins of *E. histolytica* present structural homology with TLR4 [69]. These LRRs could act as co-receptors in *E. histolytica*-bacteria interactions, although experimental evidence studying their functions in is still pending. Table 1 summarises this data.

**Table 1:** Components of the *Entamoeba histolytica* glycocalyx potentially involved in bacterial non-opsonic phagocytosis.

*Entamoeba histolytica*	Category	Potentially binding *E. histolytica* component	Proposed Bacteria component	Refs
LPPG	GPI-anchored glycoprotein	Dextran-like (Glcα1,6)_n_Gal_1_Man_2_GlcN-myoinositol	Adhesins (Gram-) PA14, PapG, Lec A (Gram+) Gbps	[34,45,46,49] [51]
Mannose-Rich Glycoproteins	N-glycosylated proteinsHgL-Lectin Gal-GalNAc	N-glycans (Man_5_GlcNAc_2_)	Adhesin FimH (Gram-)	[54,55], [32][56],
Gal/GalNAc Multimeric Lectin	Light (LgL) Heavy (HgL) Intermediate (IgL)	β-trefoil VWD/CRD CRD	LPS, CPS, LTA, Gal, GalNAc LPS, PGN, Gal, ß1–3 glucan Glycans, Prot ALPS	[18,19], [53] [60,61], [29,58]
EhGPCR-1 (TMEM-181)	7transmembrane helix	Unknown	LPS (Gram-)	[63,64]
Transmembrane kinases	TMK-39	CXXC motifs, Co-receptor	Unknown	[65,66]
LRR proteins	Leucine-rich Repeats	BspA TLR4 ectodomains	Unknown	[69]
Calreticulin	ER/chaperone	Co-receptor	Antibacterial defence	[62]

**Abbreviations:** LPPG: Lipopeptidophosphoglycan, GAG: Glycosaminoglycan,LPS: Lipopolysaccharide, CPS: Capsular polysaccharide, LTA: Lipoteichoic acid, VWD: von Willebrand-like domain, CRD: Carbohydrate recognition domain

## 6. Discussion/conclusion

The potential mechanisms of bacterial recognition facilitating non-opsonic phagocytosis by *E. histolytica* are likely multiple and reflect diverse modes of interaction: glycan-glycan, glycan-lectin, or glycoprotein-adhesin (Fig 2 and Table 1). Despite the apparent simplification of N-glycosylation, *E. histolytica* retains functional surface glycoproteins and successfully adapts to its host's environment. In the context of interactions between *E. histolytica* and bacteria, the evolutionary persistence of a simplified N-glycosylation pathway raises an intriguing comment: the predominance of surface-exposed Man_5_GlcNAc_2_ structures (and other mannose rich surface components) could provide sufficient molecular determinants for binding to bacterial adhesins. Conversely, it suggests that the elaboration of complex N-glycans at the cell surface may not be necessary for bacterial recognition in the context of non-opsonic phagocytosis. This hypothesis supports the idea that mannose-containing glycans could serve as a key molecular receptor for bacterial recognition by *E. histolytica*. Indeed, a limited set of surface-exposed mannosylated glycans or proteins could constitute an energetically economical and evolutionarily stable solution for mediating interactions of *E. histolytica* with various bacterial species.

A preliminary and speculative question thus arises: *Is the presence of mannose-containing compounds exposed on the trophozoite surface a necessary and sufficient condition for efficient non-opsonic phagocytosis by E. histolytica?* In the absence of experimental proofs to solve this question and considering the diversity of bacterial surface compounds as Gal-rich adhesins, LPS, PNG, LTA, and WTA we propose a working model concerning *E. histolytica* - bacteria interaction in function of the following premises:

1. Amoebic LPPG encounters bacterial adhesins whose domains recognise its dextran-like structure or the terminal Gal of its glycans. The specificity of this interaction—according to the type of adhesin—is expected: PA14 for Gram-negative bacteria and Gbp for Gram-positive bacteria; and for Gal-glycans the adhesins LecA or PapG (Gram-negative).

2. Amoebic surface components containing mannose - present in LPPG, HgL or other proteins - are targeted by bacterial adhesins such as FimH (Gram negative).

3. The b-trefoil conformation of LgL, VWD / CRD domains, and N-glycans of the Gal/GalNAc lectin bind glycoconjugates from various bacteria.

Studying these modes of interaction and determining their functional hierarchy in non-opsonic phagocytosis requires the implementation of powerful approaches, some of which already exist and are presented below.

## 7. Future studies for the characterisation of glycoconjugates in *E. histolytica* required for non-opsonic phagocytosis

Studies on non-opsonic phagocytosis in *E. histolytica* need to overcome the limited knowledge of the amoebic surface components involved in this process despite the diversity of the bacterial glycome. According to our models, the first molecules of choice include FimH, specific domains of diverse adhesins, and LPS from the bacteria counterpart. From the *E. histolytica* side, LPPG and the Gal/GalNAc lectin stand out. The combination of technologies—such as glycan microarray platforms, bioinformatics, deep learning, microfluidics, high-resolution microscopy, and image data analysis—with appropriate living models (e.g., 3D intestinal models, colon explants) will provide new insights into the field of parasite glycobiology.

### 7.1. Glycan arrays and computational glycobiology

Glycan arrays enable high-throughput analysis of glycan–protein interactions and can be applied to study adhesin binding to LPPG/dextran-like structures or the Gal/GalNAc lectin. These arrays have already identified glycans bound to FimH [70]. Computational modelling has significantly advanced the prediction of protein–glycan interactions, supporting the development of quantitative computational glycobiology [71,72]. Beyond arrays, deep learning, and bioinformatics approaches, as well as large glycan sequence datasets, further facilitate the study of host–microbe interactions, including bacterial phagocytosis, immunogenicity, pathogenicity, and glycan-mediated immune evasion [72].

### 7.2. Glycocalyx imaging and images analysis

Glycocalyx imaging has faced major challenges due to the dense, sub-10 nm spacing of glycans and the lack of suitable high-resolution, structure-preserving techniques. Genetic tagging is inapplicable because glycans are secondary gene products, whereas lectin-based labelling suffers from low affinity and insufficient specificity [73]. The identification of cell-specific glycan structures has led to the implementation of innovative—albeit complex—technological combinations, such as the use of matrix-assisted laser desorption/ionisation mass spectrometry imaging (MALDI-MSI) coupled with co-detection and digital pathology image indexing [74]. However, the ability to visualise the molecular architecture of the glycocalyx has remained challenging. A recent breakthrough in microscopy combines resolution enhancement by sequential imaging (RESI) with metabolic labelling, enabling visualisation of individual sugars within glycans on cell surfaces. This approach achieves an unprecedented 9 Å resolution in optical microscopy, marking a transformative advance for glycocalyx studies [75]. To gain mechanistic insights into how glycans and adhesins interact with LPPG/dextran-like structures or the Gal/GalNAc lectin, RESI, combined with anti-LPPG or anti-Gal/GalNAc antibodies, can be used to quantify FimH or LPS binding to the glycocalyx of *E. histolytica*. The power of existing software (e.g., Icy [76] and the statistical method SODA (Statistical Object Distance Analysis), which uses either micro- or nano-scopy, will improve our knowledge of the spatial co-localization of engaged interactive molecules [77].

### 7.3. 3D Intestinal models to investigate amoeba-bacteria crosstalk

Although insufficiently considered, the microbiota plays a major role in the relationship of *E. histolytica* with the intestine. The influence of non-opsonic phagocytosis in the lifestyle and pathogenicity of *E. histolytica* can be studied thanks to the development of 3D intestinal models closer to the native intestine [78]. For example, gut-on-a-chip systems demonstrate that peristalsis accelerates tissue invasion by *E. histolytica* [78]. This Entamoeba-3D intestinal model can be challenged with commensal microbes to determine the role of identified surface bacterial factors in the onset of amoebiasis.

Overall, these technologies offer real opportunities to discover the role of glycans on bacteria non-opsonic phagocytosis of *E. histolytica*. Advances in the analysis of the amoebic glycocalyx will be essential to better understand glycoconjugate–bacteria interactions and amoebiasis

## Abbreviations

aa: amino acid
BspA: Bacteroides surface protein A
CDT: cytolethal distending toxins
CPS: Capsular polysaccharides
CRD: carbohydrate-recognition domain
DIC: differential interference contrast
ER: endoplasmic reticulum
GAGs: glycosaminoglycans
Gbps: Glucan-binding proteins
LLO: lipid-linked oligosaccharide
LRR: Leucine-Rich Repeat
LTA: lipoteichoic acids
PAMPs: Pathogen-Associated Molecular Patterns
RESI: resolution enhancement by sequential imaging
TLRs: Toll-like receptors
TMKs: Transmembrane Kinases
VWD: von Willebrand-like domain
WTA: wall teichoic acids.
